# First nosocomial infections in children supported by veno-arterial extracorporeal membrane oxygenation (VA-ECMO)

**DOI:** 10.1186/s12887-023-03908-3

**Published:** 2023-02-23

**Authors:** Vladimir L. Cousin, Robert Rodriguez-Vigouroux, Oliver Karam, Peter Rimensberger, Klara M. Posfay-Barbe

**Affiliations:** 1 Pediatric Intensive Care Unit, Department of Pediatrics, Gynecology and Obstetrics, Geneva University Hospitals, Geneva, Switzerland; 2 Geneva University Hospitals, Geneva, Switzerland; 3 Section of Pediatric Critical Care, Department of Pediatrics, Yale School of Medicine, New Haven, CT USA; 4 Pediatric Infectious Diseases Unit, Department of Pediatrics, Gynecology and Obstetrics, Geneva University Hospitals, Geneva, Switzerland

**Keywords:** ECMO, PICU, Infection, Nosocomial

## Abstract

**Background:**

Veno-arterial Extracorporeal Membrane Oxygenation (VA-ECMO) is a standard procedure for patient with refractory shock in Pediatric Intensive Care Unit (PICU). There is a paucity of data on the time relationship between VA-ECMO support, nosocomial infection occurrence, and PICU length of stay (LOS). The aim of this study was to determine the characteristics and impact of ECMO-related infections.

**Methods:**

This is a retrospective study from 01/2008 to 12/2014, enrolling children with a VA-ECMO support for > 6 h. We recorded the first PICU infection during the VA-ECMO run, defined as a positive microbiological sample with clinical signs of infection or clinical signs of severe infection without positive sample.

**Results:**

During the study period, 41 patients (25/41 male) were included, with a median age of 41.2 months (IQR 12.9–89.9) and a 53% mortality rate. Median time on VA-ECMO was 4.2 d (IQR 2–7.1), median PICU LOS was 14.7 d (IQR 4,7–26,9). Overall, 34% patients developed an infection, with an incidence of 60/1000 VA-ECMO days. Median time to first infection was 4 d (IQR 3–5), with *Pseudomonas spp*. being the most commonly detected microorganism (42%). Infected sites were ventilator-associated pneumonia (9/14), sternotomy infection (2/14), bloodstream (2/14) and urinary tract infections (1/14). Longer VA-ECMO support (> 5 d) (OR 5.9 (CI 95% 1.4–24.6; *p* = 0.01) and longer PICU stay (> 14 d) (OR 12 (95% CI 2.2–65.5; *p* = 0.004) were associated with infection.

**Conclusion:**

In this single-center study, we underlined the high proportion and early occurrence of infections in patient on VA-ECMO, mostly in the first week. As infection was an early event, it may prolong the duration of VA-ECMO support and PICU LOS. Further research is needed to better understand the impact of infections on VA-ECMO and develop prevention strategies.

**Supplementary Information:**

The online version contains supplementary material available at 10.1186/s12887-023-03908-3.

## Background

Since the first reports of its use more than 40 years ago, veno-arterial Extra-Corporeal Membrane Oxygenation (VA-ECMO) has become a standard therapy in case of refractory cardiovascular failure [1, 2]. Patients requiring such support are at risk of numerous complications, such as bleeding, thrombosis or nosocomial infections [3]. Nosocomial infections are described to occur at a frequency between 16 to 42% [4–8]. Therefore, with up to a third of VA-ECMO patients developing an infection and as these patients may have a worse outcome [9], it is an important problem to address.

A review of nosocomial infections related to ECMO in pediatric patients by MacLaren et al. underlines the relative small number of studies only in the pediatric population, the lack of knowledge on the timing of infections, and the wide range of microorganisms, depending on the reporting center [3].

Therefore, in addition to determining the frequency and timing of nosocomial infections in children on VA-ECMO support, the aim of this study was to assess microbiological characteristics and to identify factors associated with VA-ECMO-related infections.

## Methods

### Design and procedure

This study was conducted in a 12-bed general PICU at the University Hospitals of Geneva, in Switzerland, from 01/2008 to 12/2014. It was approved by local Ethics Committee (CCER Commission Cantonale d’Ethique de la Recherche sur l’être humain https://www.ge.ch/ccer-obtenir-autorisation-recherche-medicale-etre-humain) (CE 14–231). After reviewing the protocol, the Ethics Committee waived the need for informed consent.

## Patients

Inclusion criteria were as follows: any children (aged 0 to 16 years) on VA-ECMO support for more than 6 h admitted to our hospital. In case of multiple episodes of VA-ECMO for the same patient, only the first ECMO use was considered for the study. The exclusion criteria were children with a primary immune deficiency. All patients were followed until death or PICU transfer or discharge.

We did not study infections occurring before VA-ECMO use and relapse of an infection that was diagnosed prior to ECMO initiation was not considered.

## VA-ECMO management

VA-ECMO cannulation was performed by cardio-vascular surgeons at the bedside or in the operating room. Use of VA-ECMO followed recommendations for management of refractory shock. VA-ECMO decannulation was discussed in a multidisciplinary team including intensivists, cardiologists, and cardiothoracic surgeons. A multidisciplinary team managed the VA-ECMO runs, which included intensivists, perfusionists, cardio-thoracic surgeons, and cardiologists. Circuits are verified 2 times daily by a perfusionist for membrane status, visible clots on the membrane, pump or tubes and cannulas. Nurses in charge of the patient also check the ECMO circuit hourly. All patients remained mechanically ventilated during the duration of VA-ECMO support. We used open systems suction the in the Unit, as such closed systems has been associated with longer mechanical ventilation duration [10].

In case of peripheral cannulation, a large central venous catheter dressing was applied directly at the insertion site. Additional dressings were applied depending on the patient and cannulas size. For central cannulas, sternum was closed by a sterile dressing, which was left in place until the time of sternal closure. Beneath the sternum dressing, mediastinal structures were protected with surgical gauzes. There was no change to this practice.

We did perform cultures in patients treated with VA-ECMO only when clinically indicated.

## Infection

We included the first infection occurring during the period of VA-ECMO support, after VA-ECMO initiation. We retrospectively reviewed all the medical records of all patients treated with VA-ECMO to determine the presence of an infection. Infections were defined as a positive bacteriological sample with clinical or biological signs of infection, or all criteria for a severe infection [hemodynamic instability (increased vasoplegia or need for increased VA-ECMO support), fever (> 38 C under VA-ECMO), low/high white blood cell (< 4 G/L or > 12 G/L), systemic inflammation (CRP > 20 mg/L)] without any positive bacterial growth [5, 9].

In case of suspected sepsis, an empiric antimicrobial therapy was initiated based on local epidemiology, which was later adapted to microbiological findings. Pediatric infectious disease specialists rounded daily on all children on VA-ECMO.

## Data acquisition

Standard demographics and clinical information were abstracted for all patients from the electronic medical record. Initial diagnosis at PICU admission was classified as refractory cardiogenic shock in the context of congenital cardiac anomaly surgery, refractory cardiogenic shock in patients with cardiac disease without surgery and miscellaneous refractory shock (cardiac arrest, refractory shock with multiple organ failure, drowning). Microbiological data were collected from the microbiology department database.

Time on VA-ECMO support, time to infection diagnosis, in-PICU mortality and PICU length of stay were also recorded.

All biological data were collected from the first day of VA-ECMO in order to standardize the data between patients. Specific biological data collected were blood glucose (mmol/L), CRP (mg/L), white blood cells (G/L), platelet count (G/L).

All bacteriological cultures and viral PCR performed during the time on VA-ECMO support were collected. Microorganisms isolated during the infectious episodes were reported. We collected data on the antimicrobial therapy at the start of VA-ECMO and any changes thereafter. Antimicrobials are reported by antibiotic class.

## Statistical analyses

Continuous variables are expressed as median and interquartile range (IQR) and categorical variable were expressed as frequency and proportion. Patients with and without infection were compared using the Mann–Whitney test, the Chi-square test or Fisher test, depending on the variables. Kaplan–Meier survival analysis was used to estimate the probability of death and the time to infection in PICU. Risk factors for infection were assessed by univariate analysis and unadjusted odds ratio (OR) and 95% confidence intervals (95% CI). Statistical significance was defined as a p value of less than 0.05. Data were analyzed using STATA 14 (StataCorp, College Station, TX, USA).

## Results

**Fig. 1:**
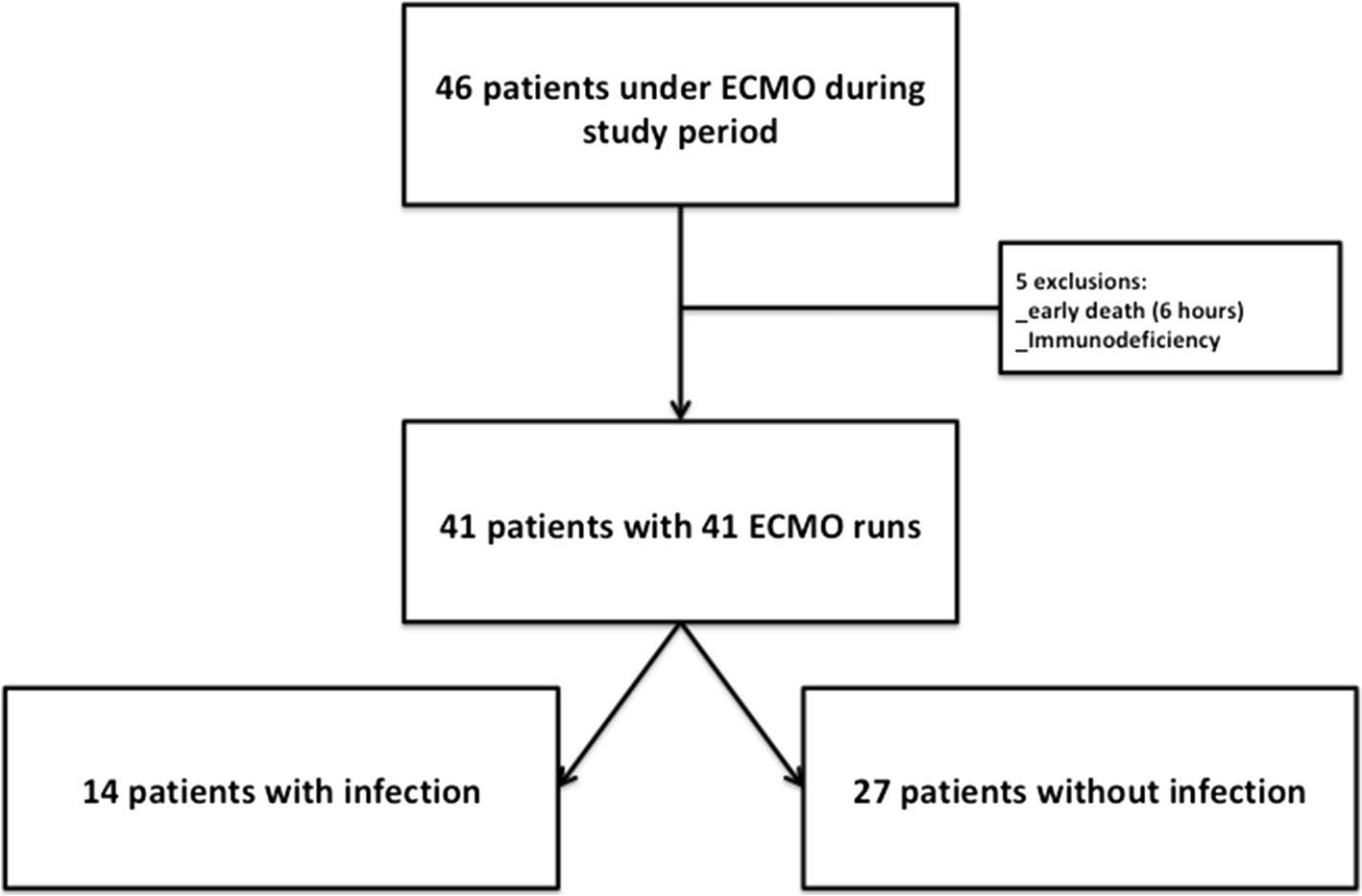
Study flowchart. VA- ECMO: venous-arterial extracorporeal membrane oxygenation

During the study period, a total of 41 pediatric patients were included (Fig. 1). The reasons for VA-ECMO support were refractory cardiogenic shock in the context of congenital cardiac anomaly surgery for 28 patients, refractory cardiogenic shock in patients with cardiac disease for 7 patients [dilated cardiomyopathy (4), pulmonary hypertension (1), previously operated tricuspid atresia with Fontan physiology (1), early post-operative infectious ARDS (1)], and miscellaneous refractory shock for 6 patients. VA-ECMO support was started in operating room, in the emergency room or in PICU in 61%, 7.3%, and 31.7% of cases, respectively. Patients were already in PICU prior to VA-ECMO initiation for 41.5% of cases, with a median length of stay of 2 days (IQR 1–4). VA-ECMO was central in 80.5% of cases, peripheral in 14.6%, and peripheral converted to central within 24 h of insertion in 4.9%.

**Fig. 2:**
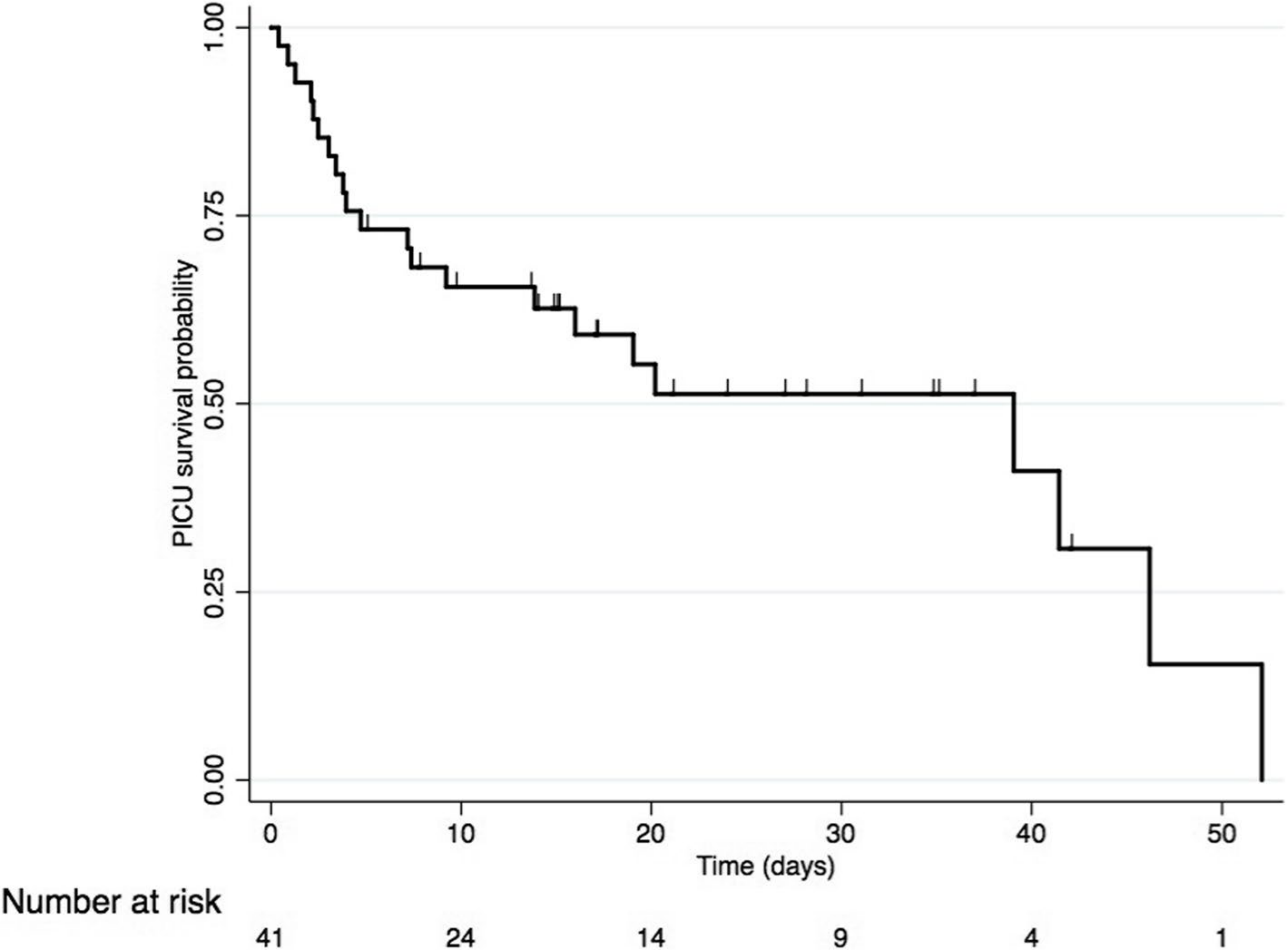
Cumulative probability of survival during PICU stay for patient with VA-ECMO. Kaplan–Meier estimated of the unadjusted cumulative probability of survival during PICU stay. Vertical lines represent censored patients (leave PICU alive). PICU: pediatric intensive care unit

Median time on ECMO support was 4.2 days (IQR 2–7.1), and median PICU length of stay was 14.7 days (IQR 4.7–26.9). Overall, mortality was 53% (22/41) (Fig. 2).

**Fig. 3:**
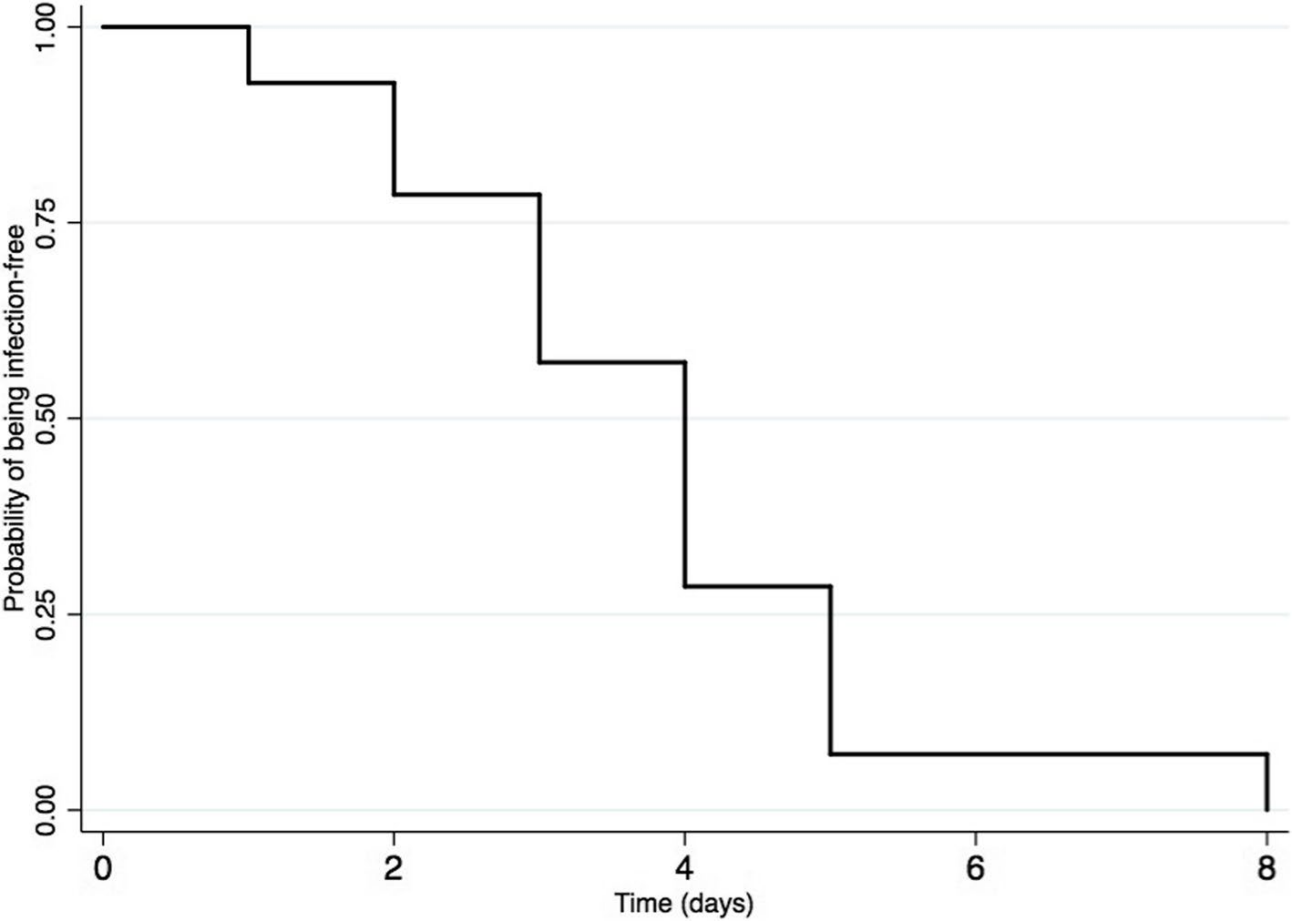
Cumulative probability of first infection during VA-ECMO support. Kaplan–Meier estimates of the unadjusted cumulative probability of being infection free for patients with infection (*N* = 14) treated with VA-ECMO

**Fig. 4:**
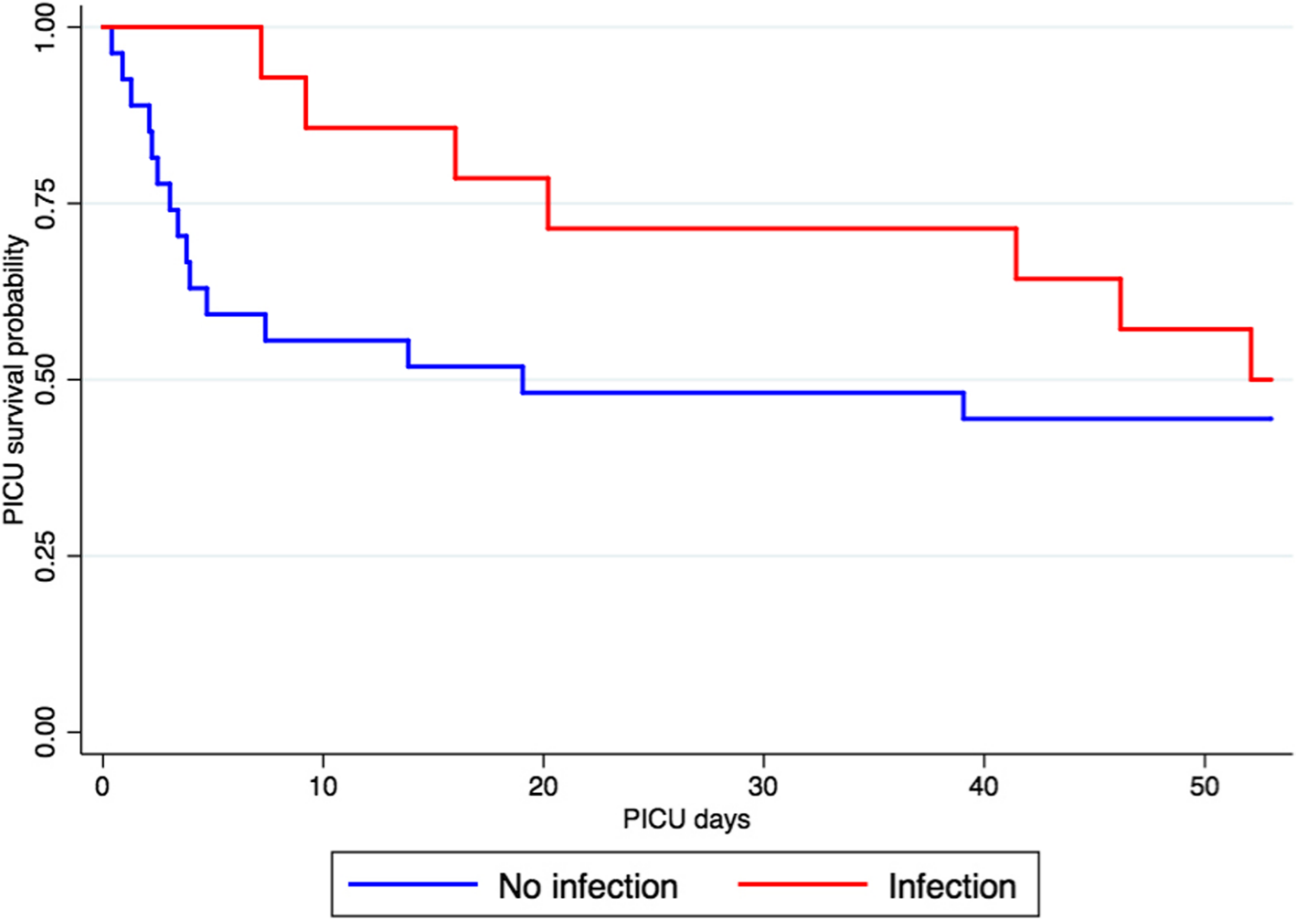
Cumulative probability of survival during PICU stay for patient with VA-ECMO, depending on infection status. Kaplan–Meier cumulative probability of survival during PICU stay for patient with veno-arterial extracorporeal membrane oxygenation (VA-ECMO), adjusted on infection status. Comparison of curve using log-rank test found significant difference with a *p* value *p* = 0.2. In blue patient without an infection (*N* = 27). In red patient with an infection (*N* = 14). PICU: pediatric intensive care unit

In this cohort, 14 cases (34%) of infections were reported with an incidence of 60.9 infections per 1000 ECMO days. Median time to first infection was 4 days (IQR 3–5); the cumulative probability of being infection free is showed in Fig. 3. Infection sites were ventilator-associated pneumonia (9/14), surgical site infection (2/14), bloodstream (2/14) and urinary tract infections (1/14). Comparison of demographic characteristics between infected and non-infected patients is summarized in Table 1. Both PICU length of stay and duration of ECMO were different between both groups. The survival probability during PICU stays between patients with and without infection was not significantly different (*p* value = 0.2; Fig. 4). However, overall mortality was not significantly different between both groups (55% versus 50%; *p* value = 0,75).

**Table 1:** Patients’ characteristics, with and without infection

	**No infection (*N* = 27)**	**Infection (*N* = 14)**	***p* value**
PICU length of stay (days)	7.7 (IQR 3–19)	24.1 (IQR 14.9–36.9)	0.002
VA-ECMO run length (days)	3.1 (IQR 1.9–6.3)	6.6 (IQR 4.5–9.4)	0.009
Age at VA-ECMO (months)	41.2 (IQR 12.9–81.8)	40.3 (IQR 9.8–112.9)	0.62
Weight (kg)	12 (IQR 7.2–20)	11 (IQR 5.7–26.8)	0.84
Hospitalized in PICU at VA-ECMO	11 (40,7)	6 (42,9)	0,9
VA-ECMO inserted in the OR	18 (66.7)	7 (50)	
VA-ECMO inserted in the ER	2 (7.4)	1 (7,1)	
VA-ECMO inserted in the PICU	7 (25.9)	6 (42,9)	0,6
Central VA-ECMO	21 (77.8)	12 (85.7)	
Peripheral VA-ECMO	4 (14.8)	2 (14.3)	
Peripheral converted to central	2 (7.4)	0(0)	0,8
Leucocytes (G/L)^a^	9.9 (IQR 5.7–13.6)	10.6 (IQR 8.2–13.3)	0.51
Platelets (G/L)^a^	69 (IQR 35–133)	74 (IQR 69–133)	0.45
CRP (mg/L)^a^	10 (IQR 10–33.5)	18 (IQR 10–76)	0.29
Blood glucose (mmol/L)^a^	7.8 (IQR 5.1–14.6)	9.8 (IQR 6.8–14.6)	0.37
Deceased	15 (55%)	7 (50%)	0.75

Data are No. (%) of patients or median (interquartile range, IQR)

^a^at day 1 of VA-ECMO support

*VA-ECMO* veno-arterial extracorporeal membrane oxygenation, *PICU* pediatric intensive care unit

Microorganisms isolated in infected patient are summarized in Table 2; the most frequent being *Pseudomonas spp*. (42%). Yeast isolation occurred in 21% of cases. Two patients had signs of infection without an isolated microorganism. At ECMO initiation, 90% of patients were treated with antibiotics. In patients infected after ECMO initiation, 92% had at least one antibiotic compared to 88% in non-infected patients. In all population, at time of positive results or after 48 h of ECMO support, ll patients but two were receiving antibiotics (95%), regardless of the infection status. All patients with an infection had an antibiotic at time of diagnosis. Complete description of antibiotic used at VA-ECMO implantation and antibiotic use is summarized in Table 3.

**Table 2:** Microorganisms associated with culture-proven infection in 14 VA-ECMO patients

Microorganisms	N (%)
*Pseudomonas spp*	6 (42)
*Haemophilus influenzae*	3 (21)
*Enterobacter cloacae*	1 (7)
*Klebsiella oxytoca*	1 (7)
*Streptococcus pneumoniae*	1 (7)
*Enterococcus faecalis*	1 (7)
Candida albicans	3 (21)
Cytomegalovirus	1 (7)
Only clinical (no germs identified)	2 (14)

**Table 3:** Antibiotic used at VA-ECMO implantation and during VA-ECMO support

**At VA-ECMO implantation**	
No antibiotics	4(10)
Cephalosporin first generation	18 (44)
Glycopeptide only	7 (17)
Glycopeptide + aminoglycoside	4 (10)
Glycopeptide + carbapenem	5 (12)
Ureidopenicillin	2 (5)
Carbapenem	1 (2)
**Adaptation during ECMO support**	
No change or adjunction/ stop	21 (52)
Cephalosporin - first generation	2 (5)
Glycopeptide only	4 (10)
Glycopeptide + aminoglycoside	4 (10)
Glycopeptide + carbapenem	2 (5)
Glycopeptide + Cephalosporin- third generation	1 (2)
Carbapenem	1 (2)
Aminoglycoside	3 (8)
Cephalosporin- third generation	1 (2)
Ureidopenicillin	1 (2)
Macrolide	1(2)

The factors associated with infection acquired on VA-ECMO were PICU length of stay and VA-ECMO duration (Supplementary Fig. 1). In order to quantify the impact of VA-ECMO duration and PICU length of stay, we separated both variables in 2 groups (more than the variables median in general population). VA-ECMO duration of less than 5 days and equal/more than 5 days were compared. PICU length of stay was compared between less than 15 days and equal/more than 15 days. In both cases, we found an association between infection and longer VA-ECMO support (OR 5.9 (CI 95% 1.4–24.6; *p* = 0.01) and longer PICU stay (OR 5 (95% CI 1.2–20.4; *p* = 0.02).

## Discussion

In this study, we described the epidemiology of infectious complications in children treated with VA-ECMO. Our results underlined the early timing of nosocomial infection in this population. Our study highlighted the association between the occurrence of infection and the duration of VA-ECMO and PICU length of stay.

A review by MacLaren et al. found an infection rate between 16 to 42% in pediatric patients with VA-ECMO support [3]. It is noticeable that there is an important variability in this number, presumably due to differences in population and infection definition in studies. In our cohort, infections occurred in 34% of patient with an incidence of 60.9 infections per 1000 VA-ECMO days. The reported incidence by others varied between 20 to 116 infections per 1000 VA-ECMO days, and only the study by Ayyildiz et al. reported a higher incidence than our cohort [5, 7, 9, 11]. Importantly, a significant proportion of patients on VA-ECMO treated with antibiotics during VA-ECMO did not seem to have prevented the occurrence of infection. Our population may have non-measurable risk factors but also specific patient management in PICU explaining such high incidence, like the high proportion of central VA-ECMO. It should warrant us to review the management of our patients treated with VA-ECMO and review the prevention measures such as insertion protocol bundles and other infection prevention measures [12].

We found that infection occurred in the first days under VA-ECMO support an information not well described in pediatric literature. In children, O’Neil et al. showed that most infections occurred during the first week and Cashen et al. described a similar timing nearly 20 years later [4, 6]. Interestingly, in adults, two reports by de Roux et al. and Schmidt et al. found a similar timing of infection with high incidences during the first 7 days of VA-ECMO [13, 14]. Our data supports such timing as well and recommend clinicians to be particularly aware of the risk of infections even during the first days of VA-ECMO support.

The microorganisms isolated underlined the role of *Pseudomonas spp.* in our unit, which is in line with the large study by Bizzarro et al. [9]. It may be a consequence of the important, wide spectrum antibiotic use in this population. It may suggest the use, when infection is suspected, of an anti-pseudomonas antibiotic in a patient with VA-ECMO. Importantly, a *Candida spp.* was isolated three times, underling the risk of fungal infection in patient with VA-ECMO [9]. To note, fungal infection could increase the risk of unfavorable impact on patient outcome, as underlined by the study of Pluim et al. [15].

Two important points about infection in VA-ECMO patients should be discussed. First, our institutional protocol did not recommend the use of routine blood cultures. Such practice is under debate in the literature, but some interesting data suggest it could be beneficial in certain high-risk situations and it may help to identify earlier bacteriemia [13]. Second, even if our institutional protocol did not recommend antibiotic prophylaxis, it is notable that the vast majority of patients had in fact an antibiotherapy in place at VA-ECMO initiation. Use of antibiotic prophylaxis is not currently recommended by ELSO but some center used it [16, 17]. Impact on the incidence of nosocomial infections is difficult to assess but the high proportion of *Pseudomonas spp.* infection in our cohort raised the question on the impact of such high antibiotic pressure in our patients.

We found an association between the occurrence of infection and the duration of VA-ECMO and the PICU length of stay, as previously described [6–9, 11]. Longer exposition to invasive devices, such as catheters and VA-ECMO cannulas, could explain the increased risk of infection. However, it is important to note that no existing analyses are able to determine whether longer duration of support or PICU stay is in fact a risk or a consequence of infection [16]. Some sicker patients may die before they can be infected, while others may stay longer with VA-ECMO support in the PICU, remaining at risk for infection, as suggested by our results with patient infected having better earlier survival. However, infection itself might also increase the duration of VA-ECMO support and the PICU length of stay.

We are aware of several limitations of this study. First, there is currently an absence of consensual definition of VA-ECMO nosocomial infection and we used an already published definition, by ELSO [9]. Second, even with great effort to limit our study to first infection under VA-ECMO, we cannot rule out that some infections may be present before ECMO initiation. Third, the design of our study was a retrospective, single-center study with a small number of patients and specific local practice such as use of open systems suction or absence of routine blood cultures protocol. Results may be difficult to generalize. Fourth, we do not have data on nosocomial infection occurrence in other patients in the Unit, as such surveillance program only started last year in the Unit. But, as underlined by MacLaren et al. there are a relatively small number of studies in the pediatric population with a paucity of data on the timing of infections [3]. Our results may bring some interesting data to better understand the timing and risk factors of infection in patients under VA-ECMO support.

## Conclusions

Our findings indicate that pediatric patients treated with VA-ECMO are at high risk of early nosocomial infections. Occurance of nosocomial infection was associated with longer VA-ECMO support and PICU stay but infection did not impact patient survival. Future research should be performed to have more comprehensive understanding of nosocomial infections on VA-ECMO to improve their prevention and management.

## Supplementary Information

**Additional file 1:** **Supplementary Figure 1.**Detailed description of PICU length of stay and VA-ECMO duration.

## Data Availability

The dataset used and/or analyzed during the current study are available from the corresponding author on reasonable request.

## Abbreviations

VA-ECMO: Veno-arterial Extra-Corporeal Membrane Oxygenation
PICU: Pediatric Intensive Care Units
