# Supplementary material for: First nosocomial infections in children supported by veno-arterial extracorporeal membrane oxygenation (VA-ECMO)
Source: BMC Pediatr. 2023 Feb 23;23:89. doi: 10.1186/s12887-023-03908-3 (PMC9948414; doi:10.1186/s12887-023-03908-3)
Supplement: Supplementary file 1 — Additional file 1: Supplementary Figure 1.Detailed description of PICU length of stay and VA-ECMO duration. [file 12887_2023_3908_MOESM1_ESM.docx]

**Supplementary Figure 1: Detailed description of PICU length of stay and VA-ECMO duration**


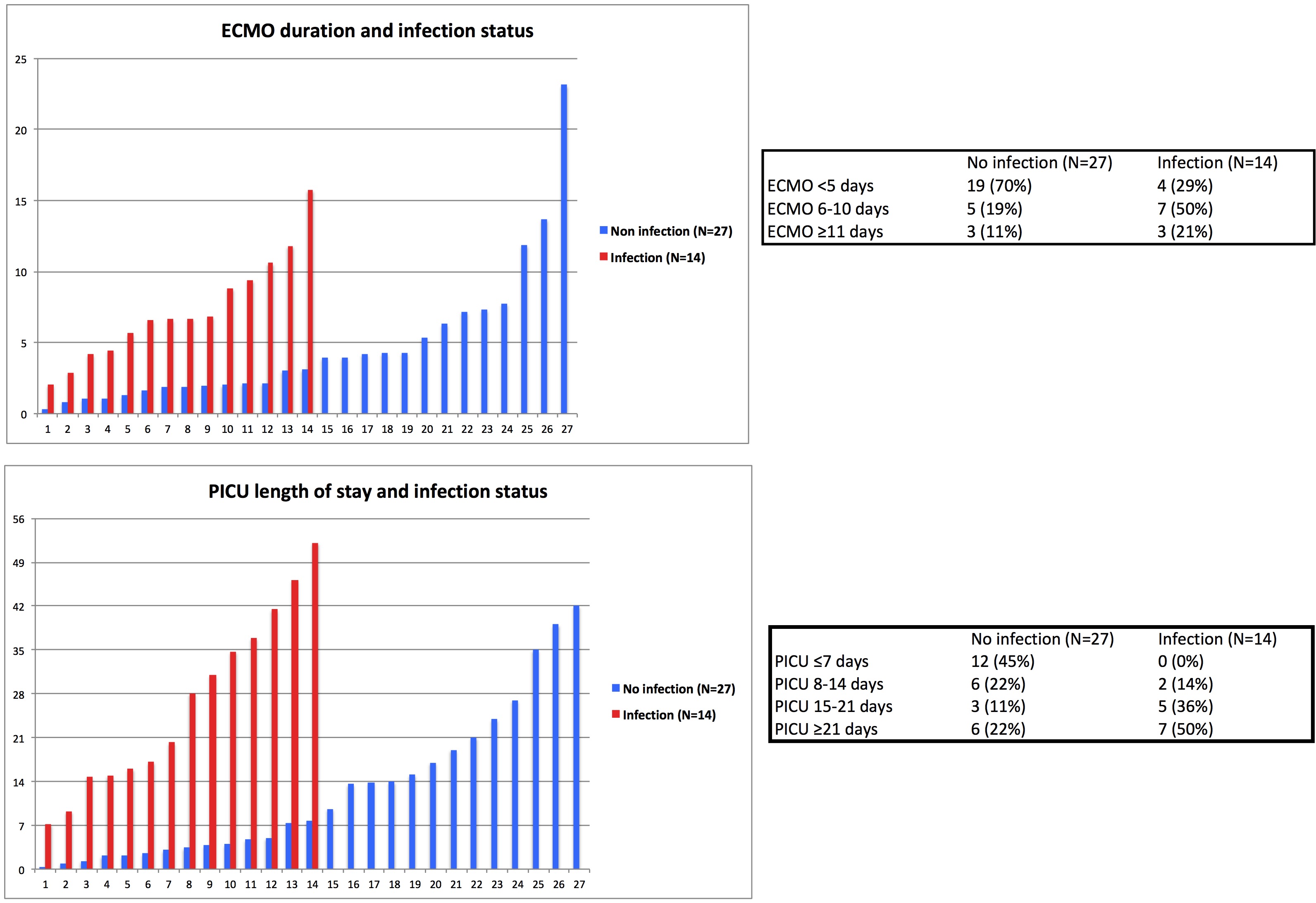


Detailed description of VA-ECMO duration in the upper panel and of PICU length of stay in the lower panel.

Upper panel : ECMO duration described per patient (blue non infected ; red infected). In the table, patients were separated in 3 categories : VA-ECMO < 5 days, 6-10 days, ≥11 days. There was a majority of infected patients in the 6-10 days and ≥11 days groups. The overall comparison was statistically significant (p value 0.036)

Lower panel : PICU length of stay, described per patient (blue non infected ; red infected). In the table, patients were separated in 4 categories : PICU length of stay ≤7 days, 8-14 days, 15-21 days and ≥21 days. There was a majority of infected patient in the last two categories. The overall comparison was statistically significant (p value 0.004)
